# Esophageal Foreign Body Removal: A Novel Approach

**DOI:** 10.7759/cureus.18081

**Published:** 2021-09-18

**Authors:** Muhammad Saad Siddique, Aqsa Mumtaz, Muhammad Subhan Saeed, Baha Aldeen Bani Fawwaz, Abu Hurairah

**Affiliations:** 1 Internal Medicine, Siddique Hospital, Sheikhupura, PAK; 2 Internal Medicine, Mayo Hospital, Lahore, PAK; 3 Internal Medicine, AdventHealth Orlando, Orlando, USA; 4 Gastroenterology and Hepatology, AdventHealth Orlando, Orlando, USA

**Keywords:** removal, endoscopy, adults, foreign body, esophagus

## Abstract

Upper esophageal foreign body impaction is a common clinical presentation and often requires medical attention. The most common foreign bodies encountered in the adult population are food-related, e.g., steak pieces and meat bones. Endoscopic interventions are indicated when the foreign objects fail to pass spontaneously. The standard methods to remove these foreign bodies include push technique and retrieval methods using various endoscopic instruments. However, we report a unique method that was used to remove a large upper esophageal impacted foreign body refractory to removal by standard procedures.

## Introduction

Upper gastrointestinal (GI) foreign body impaction is one of the leading indications for endoscopy in pediatric patients but is less common in adults. It is one of the most common indications for emergent endoscopies [1]. Most of the foreign bodies in the GI tract pass spontaneously; however, 10-20% require endoscopic maneuvers, and less than 1% require surgical procedures [2]. Most of the esophageal foreign bodies are food-related [3] and are impacted due to an underlying structural abnormality; Schatzki ring and peptic stricture of the esophagus being the most common ones [4], or an underlying motility disorder [4]. We report a case of an unusual foreign body, a large garlic clove, that was stuck at the level of the upper esophageal sphincter and was refractory to removal by standard endoscopic techniques. The normal esophageal anatomy and endoscopically normal motility in the reported patient make this case study more unique. The method that we used to remove the foreign body is unique and we propose that it can potentially be used for the removal of unusually large foreign bodies impacted in the upper esophagus.

## Case presentation

**Figure 1:**
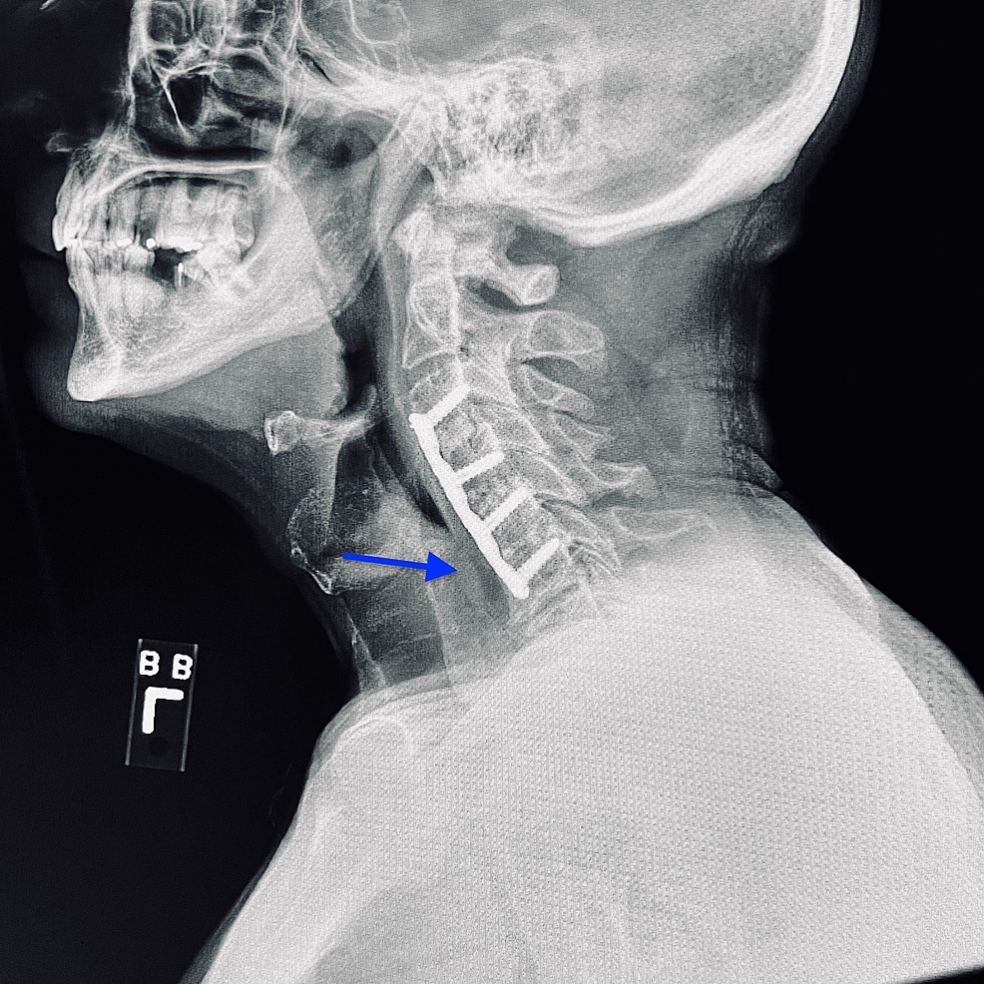
Lateral view of X-ray neck showing mild distension of the proximal esophagus (arrow)

**Figure 2:**
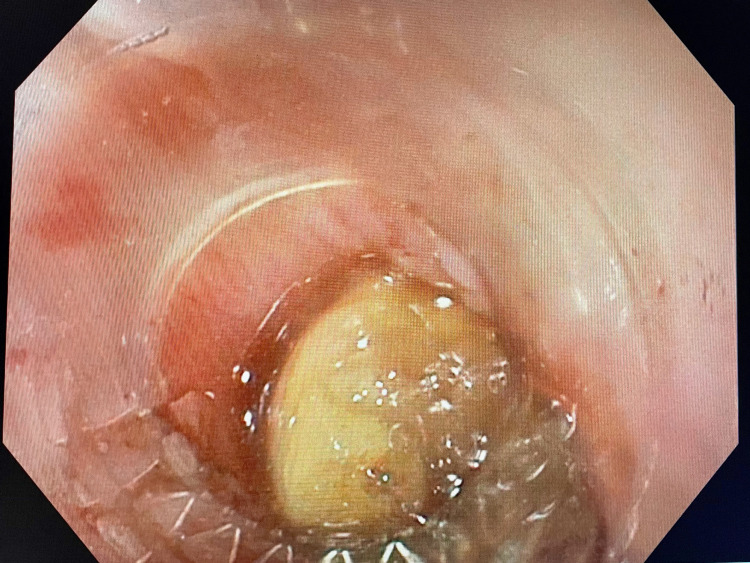
Endoscopic view of the impacted foreign body

A 57-year-old male presented to the ER with odynophagia and excessive drooling after swallowing a clove of garlic earlier that day. The patient denied any shortness of breath on presentation. He also denied any previous episodes of food impaction, dysphagia, or odynophagia. There was no prior history of upper GI endoscopy and no family history of any GI disorders. His past medical history was insignificant apart from hypertension and cervical spondylopathy status post-anterior cervical plating. The patient was hemodynamically stable with blood pressure (BP) of 139/79 mmHg, heart rate (HR) of 91 beats per minute, temperature (T) of 98 °C, and respiratory rate (RR) of 16 breaths per minute. On examination, the patient appeared to be in acute distress with an otherwise unremarkable exam. An X-ray of the neck was done in the ER, which showed mild distension of the proximal esophagus and some hypodense material in the lumen at the C6-C7 level, which could represent impacted food material (Figure 1). No radiopaque foreign body was reported, and the patient was urgently referred to the gastroenterology department. On emergent esophagogastroduodenoscopy (EGD) under general anesthesia with the airway protected by intubation, food bolus impaction was identified in the upper esophagus 15 cm from the incisors (Figure 2). The performing physician tried to dislodge it using standardized procedures including push technique, grasping forceps, and polypectomy snares, but due to the large size of the foreign body, those efforts were unsuccessful. We had a discussion as to whether an ENT consult would be appropriate or not, and our team concluded that since the patient was stable, breathing comfortably on room air, and as the foreign body was in the esophagus, this consult would not change the management plan. Therefore, the procedure was then deferred and a repeat EGD was planned for the next day.

**Figure 3:**
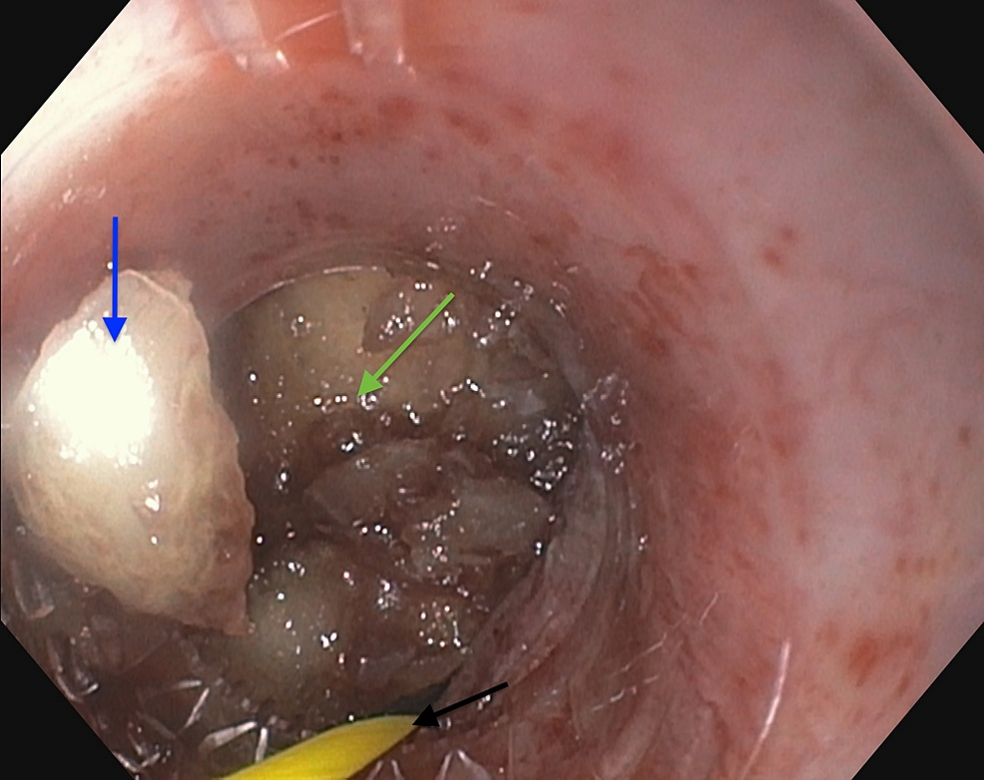
Endoscopic view of the foreign body during removal Foreign body marked with a blue arrow, soft tip Hydra Jagwire™ marked with a black arrow, and ballon marked with green arrow

**Figure 4:**
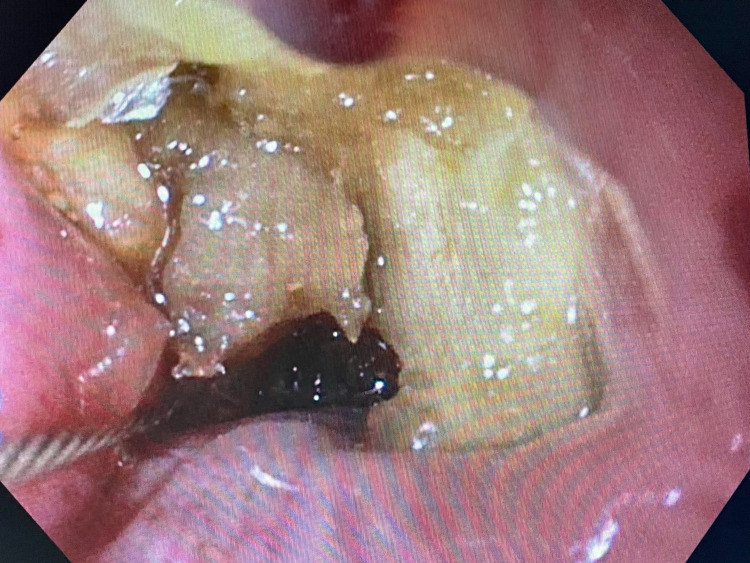
Passage of polypectomy snare to grasp the foreign body

**Figure 5:**
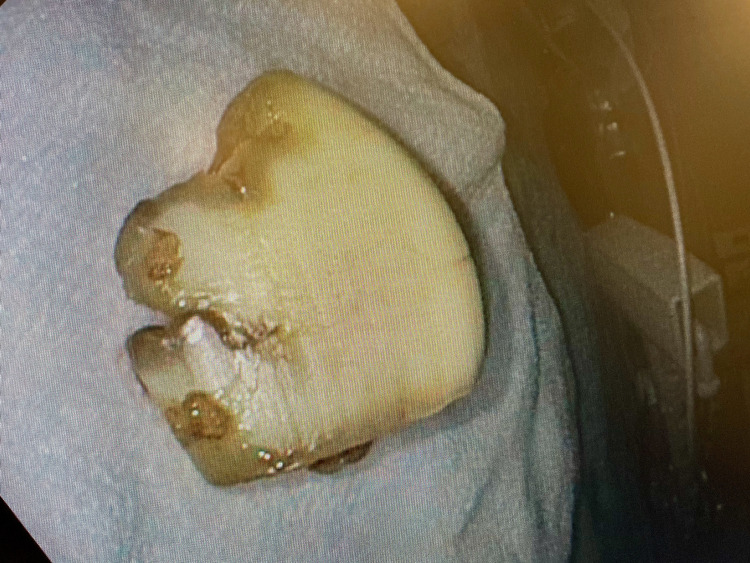
Removed foreign body (i.e., a garlic clove)

On repeat EGD under general anesthesia with intubation, which was conducted less than 12 hours from the presentation, the foreign body was seen lodged at the level of the upper esophageal sphincter (UES). Initially, a cap was attached to the end of the scope, but it could not be dislodged. Rat-tooth forceps (13 mm) were then used to grasp the food bolus with no success. Next, a Boston Scientific Hydra Jagwire™ (Boston Scientific, Marlborough, MA) was maneuvered alongside the bolus into the distal esophagus. A biliary balloon catheter was inserted into the guidewire and inflated, and the catheter was dragged up, dislodging the food bolus into the oropharynx (Figure 3). Using a large polypectomy snare, the food bolus was grasped and removed intact (Figures 4, 5). No fluoroscopy was used during the procedure. After the successful removal of the foreign body, the scope was reinserted to reexamine the oropharynx and the upper esophagus with washing and suctioning. A mild stricture and significant erythema were noted at the UES. No mucosal disruption, tears, or perforation was noted. After the procedure, the patient was stable and tolerating a soft diet, and hence he was discharged home. A follow-up EGD was done four weeks later and showed a normal-appearing esophagus, with the resolution of erythema and inflammation. The patient was advised to transition to an advance diet as tolerated after the follow-up visit.

## Discussion

The prevalence of foreign body impaction is relatively low in adults and is usually managed conservatively and left for spontaneous passage. If conservative management fails, removal with endoscopy is usually the next step. Surgery is reserved for complicated cases and cases with unsuccessful endoscopic interventions [5].

The diagnosis of GI foreign body is clinical and is primarily made based on the history, signs, and symptoms on presentation, including the timing since ingestion, subjective complaints, and nature of the foreign body. A thoroughly obtained history helps to decide the management plan [2,6-9]. Radiography, while not always required to make the diagnosis, is recommended as an initial diagnostic test [10]. Radiographs are less helpful for radiolucent objects but can certainly confirm the diagnosis and provide valuable information regarding the location, size, and nature of the radiopaque objects. Furthermore, it can also help to rule out serious complications like pneumomediastinum, etc.

Esophageal foreign bodies can either present with incomplete or complete obstruction. In incomplete obstruction, the symptoms are milder and the patient might be able to swallow liquids. In complete obstruction, patients present with the inability to swallow liquids, excessive drooling, and possible shortness of breath. Hence, patients with complete obstruction are considered high risk for aspiration and require urgent removal [11]. The European Society of Gastrointestinal Endoscopy (ESGE) recommends that the endoscopic removal of food bolus with complete esophageal obstruction should be emergent, preferably within two hours or at least within six hours due to the high risk of aspiration [12]. Standardized endoscopic techniques including grasping forceps, polypectomy snares, Dornier-type stone retrieval baskets (Dornier MedTech, Munich, Germany), retrieval snare net, transparent cap-fitting device (used for endoscopic mucosal resection) [9], and overtube [13,14] have been used to remove foreign bodies from the esophagus.

The European Society of Gastroenterology suggests that “the push technique” is reliable, safe, and has a success rate of over 90% in pushing the impacted food boluses into the stomach. This involves the use of air insufflation and gentle pushing pressure by using an endoscope. However, this technique should not be used if significant resistance is faced as it poses a high risk of esophageal perforation [12]. Furthermore, if the push technique is not successful, endoscopic retrieval by using instruments like grasping forceps, polypectomy snares, retrieval net, or Dormia basket should be attempted.

In our case, the standard endoscopic removal techniques like the push technique and retrieval methods had failed. Ultimately, the food bolus was successfully removed with the help of a soft tip Hydra Jagwire™ and a balloon. This "dislodge, drag, and grasp" technique has not been reported to be used for the removal of a large foreign body previously. Successful removal of the foreign body with this technique warrants further studies to check its efficacy. This can help avoid the need for surgical removal and its associated risks.

Li et al. (2019) have reported a similar case involving an unusually large stone (25 mm) impacted in the upper esophagus, which could not be removed by standard removal methods using alligator forceps and fiberoptic esophagoscopy with a snare. It was successfully removed with gallbladder-grasping forceps before proceeding to esophagectomy [15]. However, our technique is significantly different as we maneuvered endoscopic instruments alongside the bolus into the distal esophagus and retrieved the foreign body.

Longstreth et al. (2001) have reported that anatomic abnormalities like Schatzki rings or peptic strictures are found in 88% of the cases of esophageal foreign body impaction [4]. Subsequently, these conditions may lead to a history of multiple impaction episodes. Esophageal motility disorders like achalasia may also have a similar presentation. However, in our case, the patient was found to have no anatomic abnormality or motility disorder as confirmed on a follow-up endoscopy four weeks after the initial presentation, and he had no history of previous esophageal impaction episodes. However, we did notice that the patient was status post-anterior cervical plating as seen on the lateral X-ray of the neck (Figure 1). We believe that this could potentially cause extrinsic compression and contribute to food bolus impaction in the esophagus.

## Conclusions

Large foreign bodies are generally associated with a higher risk of perforation and hence should be removed as soon as possible. We conclude that the aforementioned method of passing an endoscopic guidewire alongside the foreign body, inflating the balloon, and later pulling it back to dislodge the foreign body can potentially be used to remove esophageal foreign bodies. This method is relatively safe and reduces the need for surgical intervention. Foreign bodies that cannot be removed by endoscopic procedures are ultimately required to be removed by thoracotomy, but we propose that the relatively less invasive technique employed in the reported case could potentially reduce the indication for such a high-risk surgical procedure.
